# Surface Solid Dispersion and Solid Dispersion of Meloxicam: Comparison and Product Development

**DOI:** 10.15171/apb.2017.068

**Published:** 2017-12-31

**Authors:** Mayank Chaturvedi, Manish Kumar, Kamla Pathak, Shailendra Bhatt, Vipin Saini

**Affiliations:** ^1^Department of Pharmaceutics, Rajiv Academy for Pharmacy, Chattikkara, Mathura, India.; ^2^Department of Pharmaceutics, M M College of Pharmacy, Maharishi Markandeshwar University,Mullana, Ambala-133207, Haryana, India.; ^3^Department of Pharmaceutics, Pharmacy College Saifai, Uttar Pradesh University of Medical sciences, Saifai, Etawah , 206130, Uttar Pradesh, India.

**Keywords:** Surface solid dispersion, Solid dispersion, Dissolution, Orodispersible tablet

## Abstract

***Purpose:*** A comparative study was carried out between surface solid dispersion (SSD) and solid dispersion (SD) of meloxicam (MLX) to assess the solubility and dissolution enhancement approach and thereafter develop as patient friendly orodispersible tablet.

***Methods:*** Crospovidone (CPV), a hydrophilic carrier was selected for SSD preparation on the basis of 89% in- vitro MLX adsorption, 19% hydration capacity and high swelling index. SD on the other hand was made with PEG4000. Both were prepared by co-grinding and solvent evaporation method using drug: carrier ratios of 1:1, 1:4, and 1:8. Formulation SSDS3 (MLX: CPV in 1:8 ratio) made by solvent evaporation method showed t_50%_ of 28 min and 80.9% DE_50min_ which was higher in comparison to the corresponding solid dispersion, SDS3 (t_50%_ of 35min and 76.4% DE_50min_). Both SSDS3 and SDS3 were developed as orodispersible tablets and evaluated.

***Results:*** Tablet formulation F3 made with SSD3 with a disintegration time of 11 secs, by wetting time= 6 sec, high water absorption of 78%by wt and cumulative drug release of 97% proved to be superior than the tablet made with SD3.

***Conclusion:*** Conclusively, the SSD of meloxicam has the potential to be developed as fast acing formulation that can ensure almost complete release of drug.

## Introduction

Dissolution of solid dosage forms in gastrointestinal fluids is a precondition for the delivery of the drug to the systemic circulation following oral administration. The parameters that predominantly influence drug dissolution are the solubility of drug and surface area of particle.^1^ An increasing problem of poorly water soluble drug requisites obtaining a satisfactory dissolution within the gastrointestinal tract that is necessary for good bioavailability. To improve the aqueous solubility of poorly water soluble drug various techniques have been utilized such as complexation with the polymer, salt formation, addition of surfactant, prodrug and others. Solid dispersion is a frequently used technique to improve the aqueous solubility of drug where one or more active ingredient(s) is uniformly dispersed in an inert water soluble carrier matrix. Amorphization of drug, improved wettability and decrease in particle size are the main mechanisms for enhanced dissolution.^2^

In spite of several advantages of solid dispersions, the water soluble carriers used for their preparation produce soft and tacky mass which is difficult to handle especially in tablet making.^3,4^ Additionally, at high concentrations such carriers may decrease dissolution due to high viscosity in the boundary layer close to the dissolving surface.^5^ These problems can be mitigated by surface solid dispersion that uses water insoluble hydrophilic carriers and the drug is deposited on the surface of carrier.^6^ Such excipients include sodium starch glycolate, crospovidone, potato starch, silicon dioxide, croscarmellose sodium, pre-gelatinized starch and microcrystalline cellulose. Drug release from these carriers depends on the porosity, particle size and surface area of the carrier. When in contact with water, the carrier immediately disperses allowing rapid release of the drug. The dissolution and bioavailability of poorly water soluble drug is expected to improve extensively by surface solid dispersion technique.^7^ This technique when coupled with product development into orodispersible tablets is expected to further enhance the solubility of the drug. The advantages of mouth dissolving dosage/ orodispersible tablets are increasingly being recognized in both, industry and academics. The increasing popularity of these dosage forms is in part owing to various factors such as fast disintegration, good mouth feels, easy to handle, easy to swallow and effective taste.^8,9^

Meloxicam a non-steroidal anti-inflammatory and anti-pyretic agent has low aqueous solubility that delays its absorption from the gastrointestinal tract. The efforts to enhance the solubility and correspondingly the dissolution are widely reported in literature. These include the solid dispersions using hydrophilic carriers,^10^ skimmed milk,^11^ PEG 4000 by dropping method^12^ poloxamer 188 using kneading method,^13,14^ PEG 6000^15^ polyvinyl pyrrolidone using solvent evaporation method,^16^ various polymers^17^ and PEG 6000 by fusion melt method.^18^ As specified these systems are constrained with the certain limitations, the present work was aimed to develop surface solid dispersions of MLX and compare it with its solid dispersion for assessing the dissolution characteristics. Secondly to develop patient friendly dosage form and evaluate it.

## Materials and Methods

Meloxicam was supplied as gift sample from Unimark Pharmaceuticals Ltd., Ahmadabad, India. Crospovidone and sodium starch glycolate were gifted sample from International Specialility Product Technologies Ltd. USA. PEG 6000 was obtained from CDH, New Delhi and N, N-dimethylformamide from Qualikems Fine Chemicals, New Delhi. Microcrystalline cellulose, mannitol and sodium saccharin were procured from Ranbaxy Fine chemicals Pvt.Ltd. Mumbai.

### Equilibrium solubility 

An excess amount of MLX was added to 25 mL conical flasks containing different amounts of carriers CPV and sodium starch glycolate in double distilled water separately. The flasks were placed in mechanical shaker at 37±0.5°C for 48 h. At the end of 48 h the samples were filtered through Whatman filter paper and analyzed spectrophotometrically at 363 nm (Shimadzu, Pharmaspec1700, Kyoto, Japan).

### In vitro adsorption 

In vitro adsorption of drug on the carriers CPV and sodium starch glycolate was analyzed by dissolving 10 mg of MLX in 100ml double distilled water. The carriers were dispersed separately into this solution and stirred continuously by magnetic stirrer at room temperature. Samples were taken at regular intervals of 0, 20, 40, 60, 80, 100 and 120 min and assayed for unadsorbed drug at 363nm. Percent drug adsorbed was determined and plotted against time.^7^

### Hydration capacity 

One gram of the carrier was placed in 10 mL pre-weighed centrifuge tubes. Sufficient distilled water was added to make up the volume to 10 mL and the suspension was shaken vigorously for 5 min. The suspension was allowed to stand for 10 min and then excess water was removed by centrifugation at 4000rpm for 10 min and tube with sediment was then reweighed.^19^ The hydration capacity was calculated by Equation 1.

Equation 1 $$\text{Hydration capacity=}\frac{\text{weight of tube with sediment - weight of empty tube }}{\text{weight of sample on dry basis}}\text{×100}$$

### Swelling studies

Water uptake and swelling index of the carriers CPV and sodium starch glycolate were determined by method reported^19^ using indigenously developed apparatus. Weighed quantity of the carrier was subjected to the graduated arm A and double distilled water was poured in graduated arm B to a level corresponding to the height of powder pile in arm A. The level of swelling medium was maintained constant during the entire experiment. The changes in the volume (cm^3^) of the sample were recorded at different time intervals up to 2 h and swelling index was calculated by the following formula (Equation 2):

Equation 2 $$\text{Percent swelling index=}\frac{\text{Final volume - Initial volume}}{\text{Initial volume}}\text{×100}$$

### Preparation of surface solid dispersion and solid dispersion

Both SSDs and SDs were prepared by co-grinding and solvent evaporation technique, using 1:1, 1:4 and 1: 8 drug: carrier ratios. In the former method, the drug with carrier was co-grounded in a glass mortar-pestle for 30 min. The mixture was sieved through mesh (# 60) and collected for further evaluation. In solvent evaporation method, the drug was dissolved in dimethylformamide followed by dispersion of carrier into it. The mixture was heated at 60°C in a thermostatically controlled water bath till the solvent was completely evaporated and the mass so obtained was kept in a desiccator until used for the further studies.

### Evaluation of SSD and SD

#### Drug content and Equilibrium solubility

SSD equivalent to 10 mg of MLX was weighed accurately and dissolved in 10 mL of methanol. The stock solution was diluted with double distilled water and analyzed spectrophotometrically. Similar procedure was used to determine the drug content of SD. The equilibrium solubility of drug in its SSD and SD forms was determined by the method described earlier.

#### In vitro dissolution

The *in vitro* dissolution studies for pure meloxicam, SSD and SD were carried out in triplicates, in USP Apparatus II using 900 mL of double distilled water at 37±0.5°C at 100 rpm. Samples equivalent to 10 mg of meloxicam were filled in capsules (size 0) and subjected to the study. Aliquots of 5 mL were withdrawn at specified time intervals of 0, 20, 40 and 60 min and filtered through Whatman filter paper. An equal volume of fresh dissolution medium was replaced to maintain the volume of dissolution medium. The filtered samples were analyzed and used to determine % cumulative drug dissolution with respect to time.

#### Statistical analysis of in vitro dissolution data 

Model independent parameters were calculated to select the optimized system. Percent dissolution efficiency (% DE) was computed to compare the relative performance of the polymers in surface solid dispersion and solid dispersions. The magnitude of % DE was computed as the percent ratio of area under the dissolution curve up to time t (yx.dt), to that of area of the rectangle described by 100% dissolution at the same time (y100xt). It was calculated by Equation 3.

Equation 3 $$\%\;DE=\frac{\int_{0}^{t}\text{y x dt}}{\text{y100 xt}}\times 100$$

On the basis of the above interpretation, best among the related group were selected for further studies.

#### Powder properties 

The SSDs and SDs were subjected to a range of powder properties determination. Angle of repose was determined using cylinder method.^20^ Apparent bulk density (ρ_b_) was determined by pouring weighed amount of powder into a 50 cc graduated cylinder. The bulk volume (*V*_b_) was noted and divided by the powder weight to get the bulk density. The tapped density was determined by subjecting the powder to 50 tapping at height of 1 inch. The tapped volume (*V*_t_) was divided by weight of the powder to get tapped density (ρ_t_). The compressibility index which is calculated as follows (Equation 4):

Equation 4 $$CompressibilityIndex=\frac{V_{b}-V_{t}}{V_{b}}\times 100$$

The value of compressibility index below 15% indicates a powder with good flow characteristics,^20^ whereas above 25% indicates poor flow. Next, Hausner ratio which is an indirect index of ease of powder flow was calculated by dividing tapped density by bulk density.

#### FTIR

Further to confirm the identity of drug FTIR studies was carried out using Fourier transform infrared spectrophotometer (FTIR-8400S, Shimadzu, Kyoto, Japan). Pure meloxicam and KBr powder was dried in hot air oven for half an hour at 50 °C, ensuring the removal of moisture. Then the drug was mixed with KBr in the ratio of 9:1 and triturated, afterwards it was exposed to infrared rays. The scanning range of 500-4000cm^-1^ was used with 1 cm^-1^ resolution to obtainthe IR spectra of this sample.

#### Differential Scanning Calorimetry 

The samples were sealed in aluminium pans and analyzed using a DSC Q-200 V 24.4 Build 116 of TA instruments, USA. Both the sample and reference (alumina) are kept at the same temperature and the heat flow required maintaining the equality in temperature was measured. 5 to 10 mg of sample was sealed in aluminium pan and analyzed using a differential scanning calorimeter focused on the melting temperatures. A scanning rate of 10°C/min from 30°C to 300°C under nitrogen purge was applied.

#### Orodispersible tablet

The tablets of both optimized SD and SSD formulations were prepared by direct compression method. The ingredients were weighed (Table 1) and except the lubricant, were mixed in a polybag for 15 min.

At the end of mixing period magnesium stearate was incorporated and mixing was continued for another 5 min. Tablets were compressed on single punch Tablet machine and evaluated.

**Table 1:** Formulation design for orodisperable tablet of meloxicam surface solid dispersions (SSDS) and solid dispersion (SDS)

**Formulation code**	**SSDS3 mixture (equivalent to 10mg drug)**	**SDS3 mixture (equivalent to 10mg drug)**	**Mannitol (mg)**	**Crospovidone (mg)**	**Sodium saccharine Flavor (mg)**	**Microcrystalline cellulose**
F1	90	_	35	_	2	q.s
F2	90	_	35	5	2	q.s
F3	90	_	35	10	2	q.s
F4	_	90	35	_	2	q.s
F5	_	90	35	5	2	q.s
F6	_	90	35	10	2	q .s

#### Tablet evaluation

#### Thickness and Hardness

For thickness determination tablets were selected randomly from each batch and thickness was measured using Vernier Caliper (Mitotoyo, Japan). The hardness of six tablets was determined using Pfizer tester (Hicon® Grover Enterprises, New Delhi, India) and the results are expressed as average ± SD.

#### Content uniformity 

Ten tablets of each formulation were crushed in a glass pestle mortar. A powder weight equivalent to 10 mg of MLX was dissolved in 100 ml phosphate buffer, pH 7.4 and filtered. One milliliter solution was diluted to 10 ml and assayed for drug content.

#### Weight variation

Twenty tablets of each batch were selected randomly and weighed. The average weight was calculated, not more than 2 of individual weight deviated from the average weight by more than the percentage as per pharmacopoeial limits (Indian Pharmacopoeia 2007) and not deviated more than twice that percentage.

#### Disintegration time 

The disintegration time of the tablets was determined in phosphate buffer, pH 6.8 at 37±0.5 °C as per IP monograph (2007) via Tablet disintegration test machine (Hicon® Grover Enterprises, New Delhi, India). Six tablets were placed on the wire mesh just above the surface of the buffer media as a disintegrating medium present in the tube of disintegration test apparatus. The time required for each tablet to completely disintegrate and all the granules to go through the wire mesh were recorded. Results are expressed as an average of three determinations.

#### Friability 

Friability of the tablets was determined using Roche Friabilator test apparatus (Hicon® Grover Enterprises, New Delhi, India). Preweighed sample of 10 tablets was placed in the Friabilator and subjected to 100 revolutions with an operating speed of 25rpm. Tablets were dedusted using a soft muslin cloth and reweighed to calculate friability. 

#### Water absorption ratio

A piece of tissue paper folded twice was placed in small petri-dish containing 6 mL of water. A tablet was put on the paper and the time required for complete wetting was recorded. The wetted tablet was then weighed. Water absorption ratio (R), was determined by using Equation 5.

Equation 5 $$R=\frac{\left(W_{a}-W_{b}\right)}{W_{b}}\times 100$$

Where, W_b =_weight of tablet before water absorption and W_a_ = Weight of tablet after water absorption

#### In vitro release 

The release profiles of meloxicam orodispersible tablets made with SSD3 and SD3 (F1-F6) were determined using the dissolution test apparatus USP II set with a paddle speed of 50 rpm. Dissolution was tested in 900 ml of phosphate buffer, pH 6.8 maintained at 37+0.5°C. An aliquot sample of 5 mL was withdrawn, at 0, 5, 10, 15, 20 and 30 min and filtered through Whatman filter paper. An equal volume of fresh medium, which was prewarmed at 37°C replaced into the dissolution medium after each sampling to maintain the constant volume throughout the test. The samples were analyzed spectrophotometrically.

## Results

The results of *in vitro* adsorption plots of MLX on CPV and sodium starch glycolate revealed similarity in the pattern of adsorption wherein the abundant free adsorption sites led to higher initial adsorption that later on slowed down. However, the extent of adsorption of MLX on CPV was slightly higher (89%) than on sodium starch glycolate (83%). This may be due to higher larger particle size of the former that provided more surface-area for adsorption of MLX.^18^ Another determinant property of the carrier was the hydration capacity that was found to be 18% for CPV and 13% for sodium starch glycolate (Table 2).

**Table 2:** Swelling index profile of Crospovidone and Sodium starch glycolate

**Time in (hr)**	**crospovidone**	**Sodium starch glycolate**
0	0	0
30	150	110
60	275	189
120	350	230

This is because CPV exhibits its action by swelling as well as wicking which is caused due to its capillary action and porosity while sodium starch glycolate does so only by swelling phenomenon.^21^ Furthermore, the equilibrium solubility of MLX was higher in presence of CPV rather than sodium starch glycolate. The study elaborated the percent enhancement solubility of the drug with sodium starch glycolate was 300% and 335% with crospovidone due to high interfacial activity of the former.

The drug content of SDs prepared by co-grinding method (SDC1 – SDC3) varied from 89.2 - 96.6 and those prepared by solvent evaporation method (SDS1 – SDS3) varied from 90.2 – 96.3 respectively while the drug content of prepared SSDs by co-grinding method (SSDC1 – SSDC3) varied from 91.9 - 96.0 and that prepared from solvent evaporation (SSDS1 – SSDS3) varied from 89.1 – 97.9 respectively.

**Figure 1:**
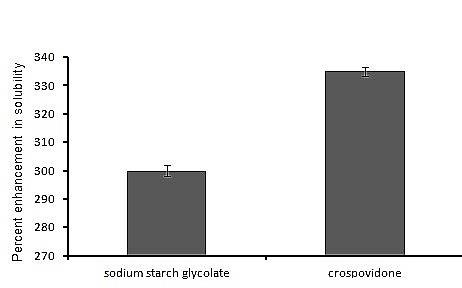
Comparative percent enhancement in solubility of meloxicam with crospovidone and sodium starch glycolate

The percentage enhancement in solubility of MLX via SSDs ranged from 58.56% – 192.66 % while the percentage enhancement in solubility of MLX via SDs was much lower in the range of 5.09% – 56.83 % (Figure 1).

**Figure 2:**
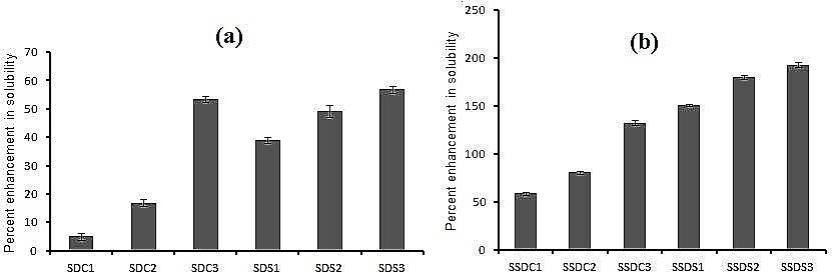
Comparative percent enhancement in solubility of meloxicam with prepared (a) solid dispersion and (b) surface solid dispersion

The pure drug showed poor dissolution characteristics in comparison to *in vitro* dissolution profiles of SSDs (Figure 2a) and SDs (Figure 2b).

Dissolution efficiency and t_50%_ were determined for SSDs and SDs and are shown in Table 3.

**Figure 3:**
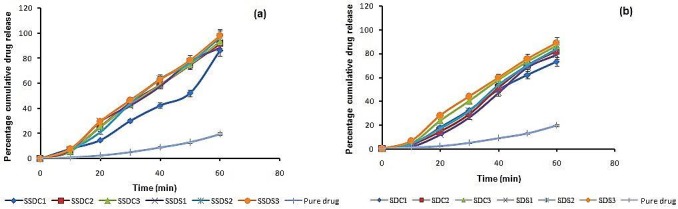
Comparative *in vitro* dissolution profile of (a) SSD’s and (b) SD’s with respect to pure drug

The powder properties of SSDS3 and SDS3 are tabulated in Table 4 and Figure 3.

**Table 3:** Model independent parameters of Surface solid Dispersion and solid dispersion

**Batch code**	**t**_50%_	**%DE**_50min_
SSDC1	48	53.1
SSDC2	35	71.7
SSDC3	32	72.1
SSDS1	38	74.8
SSDS2	45	75.4
SSDS3	28	80.9
SDC1	43	69.3
SDC2	40	71.6
SDC3	38	73.8
SDS1	45	71.5
SDS2	42	72.8
SDS3	35	76.4

**Table 4:** Micromeritics properties

**Parameters**	**SSDS3**	**SDS3**
Angle of Repose (º)	21.79±0.9	41.2±1.99
Loose density	0.325 g/mL	0.472 g/mL
Tapped Density	0.357 g/mL	0.658 g/mL
Carr´s Compressibility index	8.82%± 1.2	28.26 %±2.1
Particle Size (µm)	204.68±15.1	321.36±35.34
Hausner´s ratio	1.096±0.5	1.394±1.1

The angle of repose (21.79±0.9º) of SSDS3 was much lower than of SDS3 (41.2±1.99 º). This suggests excellent flow property of SSD (<25°) while poor flow characteristics were deduced for SDS3 that will require incorporation of flow activators in SDs in manufacturing lines. Good flow characteristics of SSDS3 can also be interpreted by low Carr’s compressibility index of 8.82%±1.2 which lies in the range for excellent particle flow (5-15%)^22^ in comparison to 28.26±2.1 for SDS3 (poor flow in 23-35). Furthermore, the Hausner’s ratio of SSDS3 was 1.09 ±0.5, which is less than 1.25 and indicated good flow property while SDS3 had a value higher than 1.25 confirming poor flow property of the latter.

**Figure 4:**
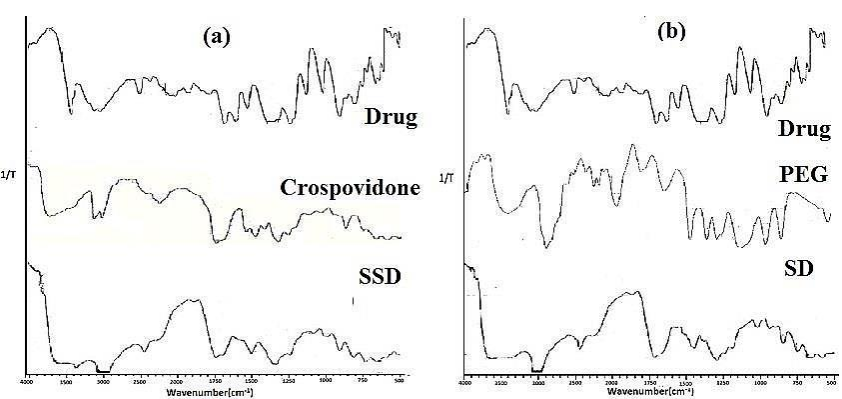
Comparative FTIR spectra of (a) Surface solid dispersion (b) Solid dispersion

The major IR peaks for MLX observed at 3136 (-N-H-stretching), 1639 (-C=O- stretching), 1280-1392 (-CN stretching), 1392-1176 (S=O stretching), 838 (-C-H-aromatic ring stretching) and were retained in SSDS3 spectrum (Figure 4).

**Figure 5:**
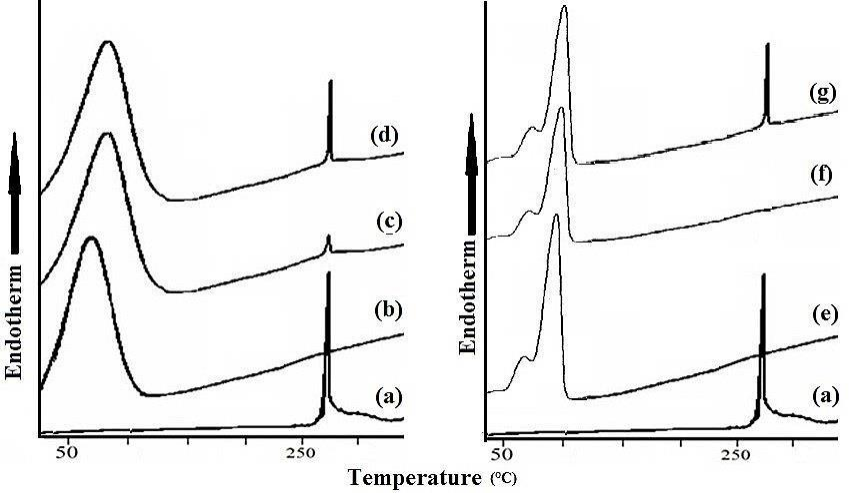
Comparative DSC analysis of (a) pure drug(Meloxicam) (b) Crospovidone (c) Surface solid dispersion (d) Physical mixture of optimized SSD (e) PEG 6000 (f) Solid dispersion (g) Physical mixture of optimized SD

The DSC thermogram of MLX (Figure 5a) showed a sharp endothermic peak at 260°C corresponding to its melting point. The thermogram of CPV (Figure 5b) exhibited a broad endothermic peak at 78.60°C with peak onset from 40.48°C. The thermogram of SSDS3 (Figure 5c) showed peaks characteristic of CPV with no additional peaks and most importantly the retention of less intense MLX peaks indicated adsorption of drug over carrier CPV. The DSC in Figure 5d and 5g referred to the physical mixture of SSDS3 and SDS3 respectively in which the peak characteristics of both drug and carriers was observed with no shifting and addition of new peaks. While that of PEG6000 (Figure 5e) showed peak characteristics at 61.5°C. The DSC of SDS3 (Figure 5f) showed peak characteristics of PEG at 60.4 with the loss of peak characteristics of drug indicating the penetration of drug inside carrier PEG6000.

Optimized formulation SSDS3 and SDS3 were developed as orodispersible tablet and evaluated. A total of six formulations were developed and evaluated for weight, diameter, thickness, hardness, friability, wetting time, disintegration time and water absorption ratio and the results are compiled in Table 5. Formulation F1, F2 and F3 weighed 148.2±1.2, 148.8±1.1 and 148.1±1.4 respectively and each having a diameter of 10.3 mm. The thickness of formulation F1, F2, F3 was 5.3 mm, 5.4 mm, 5.6 mm respectively. Hardness of tablets was measured and was found to be 3.15±0.13, 3.27±.09 and 3.32±.05 kg/cm^2^. Friability of F1 was 0.7%, F2 was 0.7% and that of F3 was 0.5%.

**Figure 6:**
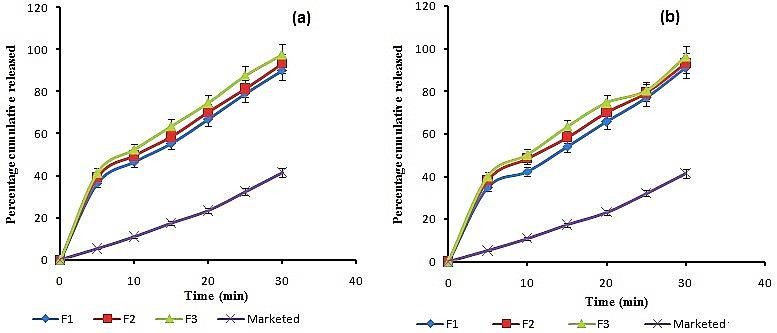
Comparative in vitro release profile of (a) SSDS3(F1- F3) orodispersible and (b) SDS3 (F4-F6) orodispersible formulation with respect to marketed formulation

*In vitro* release profiles of F1 – F6 were compared with the marketed formulation as shown in Figure 6. Formulation F3 (Figure 6a) shows 97% drug release in 30 min while marketed formulation showed only 42% drug release in 30 min.

## Discussion

*In vitro* adsorption study was aimed to evaluate the water holding capacity of the carrier materials that can affect dissolution of drug and disintegration of dosage form (in this case the tablet). Similarly, the swelling study showed 350 and 230 times swelling for CPV and sodium starch glycolate respectively in 120 h. CPV is reported as carrier with swellable adsorbent group and hence increases the solubility of poorly soluble drugs. A water insoluble but rapidly swellable synthetically cross linked homopolymer of N-vinyl-2-pyrrolidone provides efficient stearic hindrance for nucleation and crystal growth was provided by repeating units in crospovidone due to its anti-plasticizing effect. Thus, CPV with porous and granular high surface area, and high interfacial activity enhanced the solubility of MLX. Thus crospovidone with superior in vitro-drug adsorption property, hydration capacity, swelling index and solubility enhancing effect was selected for the preparation of SSD rather than sodium starch glycolate.^22^

**Table 5:** Evaluation Parameters of SSDS3 Orodispersible Tablet

**Parameter**	**F1**	**F2**	**F3**	**F4**	**F5**	**F6**
Weight (mg)	148.2±1.2	148.8±1.1	148.1±1.4	147.2±1.2	148.8±1.1	149.1±1.4
Tablet diameter (mm)	10.3	10.3	10.3	10.2	10.2	10.2
Tablet thickness (mm)	5.3	5.4	5.6	5.4	5.5	5.6
Disintegration Time (sec)	24.66±1.54	18±1.41	11.33±1.52	25.66±1.54	17±1.41	12.25±1.52
Hardness (kg/cm^2^)	3.15±0.13	3.27±.09	3.32±.05	3.45±0.14	3.47±1.0	3.48±.05
Wetting Time (sec)	14.33±0.57	11.33±058	6±0.9	18.33±0.57	15.33±058	12±0.9
Water absorption ratio (%)	65±1.3	69±2.1	78±1.77	55±1.5	60±2.2	68±1.76
Friability (%)	0.7	0.7	0.5	0.7	0.6	0.5
% Drug content	97.28	97.67	98.34	95.28	96.67	97.34

Grossly, speaking the SSDs contained insignificantly higher drug content rather than SD’s and the method of preparation had no prominent effect on drug content.

SSDs showed marked increase in solubility rather than SDs due to distribution of drug on the surface of water insoluble carriers that facilitated diffusion of drug molecules in the dissolution media readily while in SDs that drug gets entrapped inside the carrier and the release of drug molecules is hindered in comparison to SSDs. The solubility enhancement for both systems was analogous to the drug carrier ratio of 1:1<1:4<1:8 due to better wettability with increased drug carrier ratio. Furthermore, solvent evaporation method employed for preparing SSDs and SDs showed higher enhancement in solubility than co-grinding method employed. Solvent evaporation method is advantageous over co-grinding method, since evaporation of solvent leads to finer amorphization of drug particles on the carrier that increases the interfacial area of contact between the drug particles and dissolution medium.^22^

SSD’s showed enhanced dissolution characteristics in comparison to SD’s as in SSD’s water insoluble carriers were used which become hydrated in presence of water and get rapidly swell by water intake. Thus the dissolution got enhanced as the drug particles adsorbed on the carriers get wet and dissolve readily while in SD’s penetration of drug inside carrier leads to decrease in dissolution characteristics when compared with SSD’s. SSDS3 showed maximum dissolution among SSDs and SDS3 amongst SDs co-relatable to higher amount of carrier in each category. These results are in good agreement with the results obtained with equilibrium solubility studies that demonstrated enhancement in solubility on increase in the concentration of carrier.

Clearly SSDS3 showed minimum t_50%_ of 28 min and maximum %DE_50min_ of 80.9% which among all SSDs while best performing SDS3 showed t_50%_ of 35min and %DE_50min_ of 76.4% among the SDs.

Though SSDS3 affirmed superiority, SDS3 was also selected for development of tablet formulation to analyze the effect of formulation variables on the performance, if any. Higher dissolution capacity of SSDS3 can be explained by analyzing the mechanisms involved. In surface solid dispersion the drug gets adsorbed on the surface of carrier and when the carrier swells enormously, it releases the drug molecules in the release medium quickly, but in case of solid dispersion; molecular/ particulate matrix is formed between drug and carrier.^21,23^ When in dissolution medium, the water soluble carrier is released from the matrix initially followed by drug molecules, thus slow dissolution is observed in comparison to SSD. In the present study, the SSD made with crospovidone had high swelling index that resulted in the breaking of the crust layer formed by the adsorbed drug molecules resulting in fracture formation of crust. This resulted in increased rate of release of drug molecules adsorbed over carrier.

All Micromeritics results demonstrate good powder properties of SSDS3 over SDS3. The obvious reason is the use of water insoluble carrier in SSD that do not produce soft and tacky powder as seen with SDs. This is definitely advantageous aspect in the manufacturing facilities.

MLX crystals appeared to be entrapped into the particles of the carrier. FT-IR results indicate no evidence of chemical interactions between the drug and carrier crospovidone). Similarly, the signals of drug at 1689(-C=O- stretching), 1176 (symmetric S=O stretching), 1278 (-CN stretching of drug), 838 (-C-H-aromatic ring stretching) were recorded in SDS3 evidenced absence of chemical interaction between MLX and PEG6000. DSC indicated the mechanistic difference in the formation of SSD and SD using two different carriers CPV and PEG 6000 respectively. Thus, from DSC of physical mixtures, it can be concluded that drug and carrier in both SD and SSD showed no interaction.

The comparison was also done by taking three formulations made from SDS3 solid dispersions and it was observed that formulation F4–F6 showed 81-89% drug release within 30 min. which is much higher than marketed formulation but smaller than formulation F1-F3. So formulation F3 with 10 mg drug equivalent SSDS3 formulation with highest amount of CPV showed maximum release of drug among all six and marketed formulation, and had suitable properties for formulation of fast dissolving orodispersible tablet of meloxicam.

## Conclusion

Comparing SSD and SD of meloxicam, SSD showed higher dissolution enhancement than SD and the effect was extrapolated to the orodispersible tablets also. Hence SSD proved to be an important tool to enhance the dissolution rate of poorly water soluble drugs. SSD can be defined as a variant of solid dispersion but there is mechanistic difference between these two and SSD can be considered advantageous. Hence, surface solid dispersion technology can be successfully utilized for product development of drugs exhibiting dissolution rate limited absorption. 

## Acknowledgments

The authors are thankful to Torrent Pharmaceutical Ltd. Ahmadabad, India for providing gift of drug sample. We are grateful to financially supported by Rajiv Academy for Pharmacy, Mathura, UP, India.

## Ethical Issues

Not applicable.

## Conflict of Interest

Authors declare no conflict of interest in this study.
